# Supplementary material for: Meta-analysis methods for combining multiple expression profiles: comparisons, statistical characterization and an application guideline
Source: BMC Bioinformatics. 2013 Dec 21;14:368. doi: 10.1186/1471-2105-14-368 (PMC3898528; doi:10.1186/1471-2105-14-368)
Supplement: Additional file 1 — Supplementary methods of (1) Adaptive weighted (AW) Fisher, (2) Combined statistical estimates (effect size) methods of FEM and REM, (3) Combined rank statistics methods: Rank Product (RankProd) and Rank Sum (RankSum). Figure S1. MetaQC. Figure S2. Heatmap of simulated example (red color represents up-regulated genes). Figure S3. The histograms of the true number of DE studies among detected DE genes under FDR = 5% in each method for discordance case. Figure S4. Cumulative moving average to determine D = 100. Figure S5. The ROC curves and AUC for the hypothesis settings of HS A -type and (red line) HS B -type (black line) in each meta-analysis method. Figure S6. Multidimensional scaling (MDS) plots of individual data sets. Figure S7. Stability and Robustness plot for α = 0.0001, 0.005 and 0.01. Table S1. Detailed data sets description. Table S2. MetaQC results. Table S3. Data sets and number of matched genes. Table S4. Mean standardized rank (MSR) and aggregated standardized rank (ASR) for detection capability. Table S5. Mean standardized rank (MSR) and aggregated standardized rank (ASR) for biological association. Table S6. Mean standardized rank (MSR) and aggregated standardized rank (ASR) for stability. Table S7. Mean standardized rank (MSR) and aggregated standardized rank (ASR) for robustness. [file 1471-2105-14-368-S1.pdf]

Supplementary methods

Adaptive weighted (AW) Fisher

For Fisher, Stouffer and minP methods targeted on HS_B, candidate markers differentially expressed in one or more studies are detected with no indication of which studies are involved in differential expression. For example, Fishers method gives the same statistical significance for gene A with p-values= $(0.1, 0.1, 0.1, 0.1)$ and gene B with p-values= $(0.0001, 1, 1, 1)$; the two genes, however, have very different biological interpretations. The adaptively weighted Fishers method (AW) was developed to improve biological interpretation and statistical power. AW considered a weighted Fisher score $U(w_1, ..., w_k) = -2\sum_k w_k \cdot \log p_k$ (where weight w_k equals 0 or 1) and the test statistic was defined as the smallest p-value of all $2^k - 1$ possible weighted Fisher score (i.e. $T^{AW} = \min_{w_1,...,w_K} p(U(w1, ..., w_K)))$, where $p(U(w_1, ..., w_K))$ is the p-value of $U(w_1, ..., w_k)$. The resulting best adaptive weight (i.e. $W^* = \arg\min_{w1,...,w_K} p(U(w_1, ..., w_K)))$ provides indication of which studies contribute to the statistical significance of meta-analysis. For example, $w^* = (1, 1, 1, 1)$ for gene A shows statistical significance in all four studies and $w^* = (1, 0, 0, 0)$ for gene B shows statistical significance in only the first study. AW method is admissible under classical two-sample Gaussian scenario and it generally has better statistical power than traditional Fisher and minP methods in various kinds of alternative hypothesis in HS_B. For more details, refer to Li and Tseng (2011).

Combined statistical estimates (effect size) methods: FEM and REM

The meta-analysis method by combining effect sizes from several studies is a t-test based modeling approach. The effect size for a certain gene in the ith study, and $i = 1, 2, ..., K$ is defined as $d_i = \frac{\bar{T}_i - \bar{C}_i}{S_i}$, where $\bar{T}_i$, $\bar{C}_i$ and S_i denote the means of treatment and control group and the estimate of the pooled standard deviation, respectively. An unbiased estimate for d_i is obtained as $d_i^{'} = d_i - 3d_i/(4(n_i - 2 - 1))$ and the estimated variance of the unbiased effect size is $\hat{\sigma}^2_{d_i} = (n_{it}^{-1} + n_{ic}^{-1}) + d_i^2((n_{it} + n_{ic}))^{-1}$, where $n_i = n_{it} + n_{ic}$ is the sample size in the ith study; n_{it} and n_{ic} are the sample sizes of treatment and control group in the ith study respectively. From the number of studies k, a hierarchical model is given as

$$d_i = \theta_i + \varepsilon_i, \varepsilon_i \sim N(0, s_i^2)$$
$$\theta_i = \mu + \delta_i, \delta_i \sim N(0, \tau^2)$$

where s_i^2 is the variance within certain study k; τ^2 is the variance (random effect) between studies and μ is the overall mean, which is the parameter of interest. d_i and s_i^2 given by $d_i^{'}$ and $\hat{\sigma}_{d_i}^2$ are described above. $\tau^2 = 0$ means that there is no variance between studies, hence the hierarchical model reduces to a fixed effects model (FEM), $d_i = \mu + \varepsilon_i$, $\varepsilon_i \sim N(0, s_i^2)$. Otherwise, the hierarchical model is a random effects model (REM), $d_i = \mu + \delta_i + \varepsilon_i$, $\varepsilon_i \sim N(0, s_i^2)$ and $\delta_i \sim N(0, \tau^2)$. The $\hat{\tau}^2$ can be estimated by a method proposed by DerSimonian and Laird, 1986.

Combined rank statistics methods: Rank Product (RankProd) and Rank Sum (RankSum)

Rank Product (RankProd) and Rank Sum (RankSum) methods are based on a biological common sense that if the same gene is repeatedly at the top of the list ordered by up- or down-regulated genes in replicate experiments, the gene will be more likely to be regarded as differentially expressed. Suppose there are n studies with (n_{iT}, n_{iC}) replicates, $i = 1, 2, ..., k$. Below is the algorithm of finding up-regulated differential genes from Rank Product method proposed by Hong et al (2006). In the beginning, the pair-wise ratios within each study of their fold-changes were calculated (i.e., for study i, T_{ij}/C_{il}, $j = 1, 2, ..., n_{iT}$, $l = 1, 2, ..., n_{iC}$, and form $k_i = n_{iT} \times n_{iC}$ comparisons:

(1) Define the statistic of rank product $RP_g^{up} = (\Pi_i \Pi_k r_{g,i,k}^{up})^{1/k}$, where $k = k_1 + k_2 + \cdots + k_n$, and $r_{g,i,k}^{up}$ is the position of gene g in the list of genes in the ith study under k^{th} comparison sorted by decreasing pair-wise ratios calculated before.

(2) Do permutations in each array independently for B times and calculate the statistics $RP_g^{up(1)}$, $RP_g^{up(2)}$, ..., $RP_g^{up(B)}$, the same in step (1).

(3) The permutation p-value and FDR assessed by permutation within each gene can be obtained by

$$p_g = (1/GB)\Sigma_b \Sigma_g I(|RP_g^{up(b)}| \leq RP_g^{up})$$

$$FDR_g = \frac{(1/B)\sum_b \sum_g I(|RP_g^{up(b)}| \leq RP_g^{up})}{\sum_g I(|RP_g^{up(b)}| \leq RP_g^{up})}$$

In rank sum (RankSum) method, the statistic $RS_g^{up} = (\sum_i \sum_k r_{g,i,k}^{up})^{1/k}$ was used to replace the statistic RP_g^{up} from the algorithm of rank product (RankProd) mentioned above. This method only considers gene ranks

rather than absolute expression values, which leads to its robustness against heterogeneity across different studies.

Supplementary Figures

Supplementary Tables

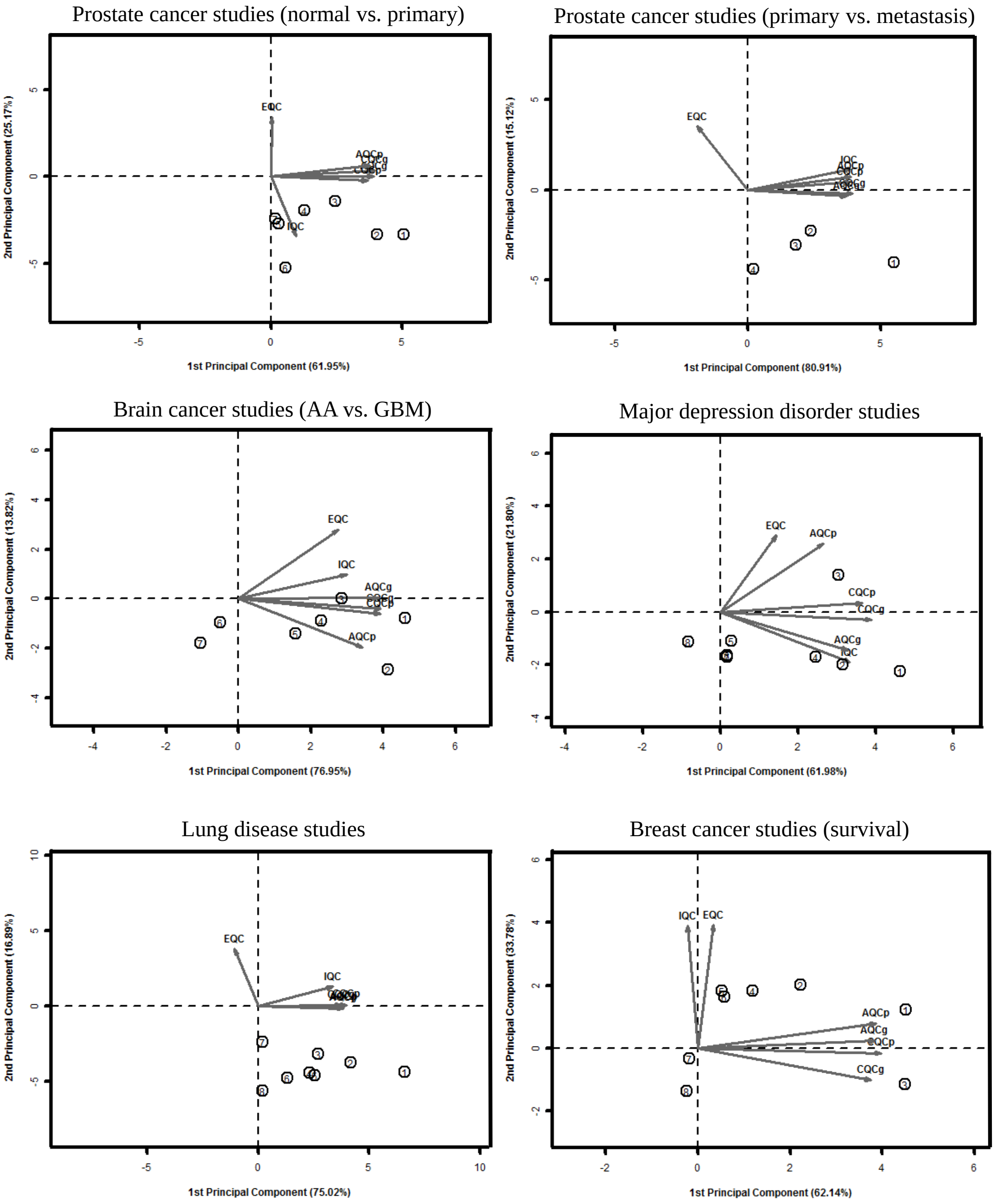

Figure S1: MetaQC

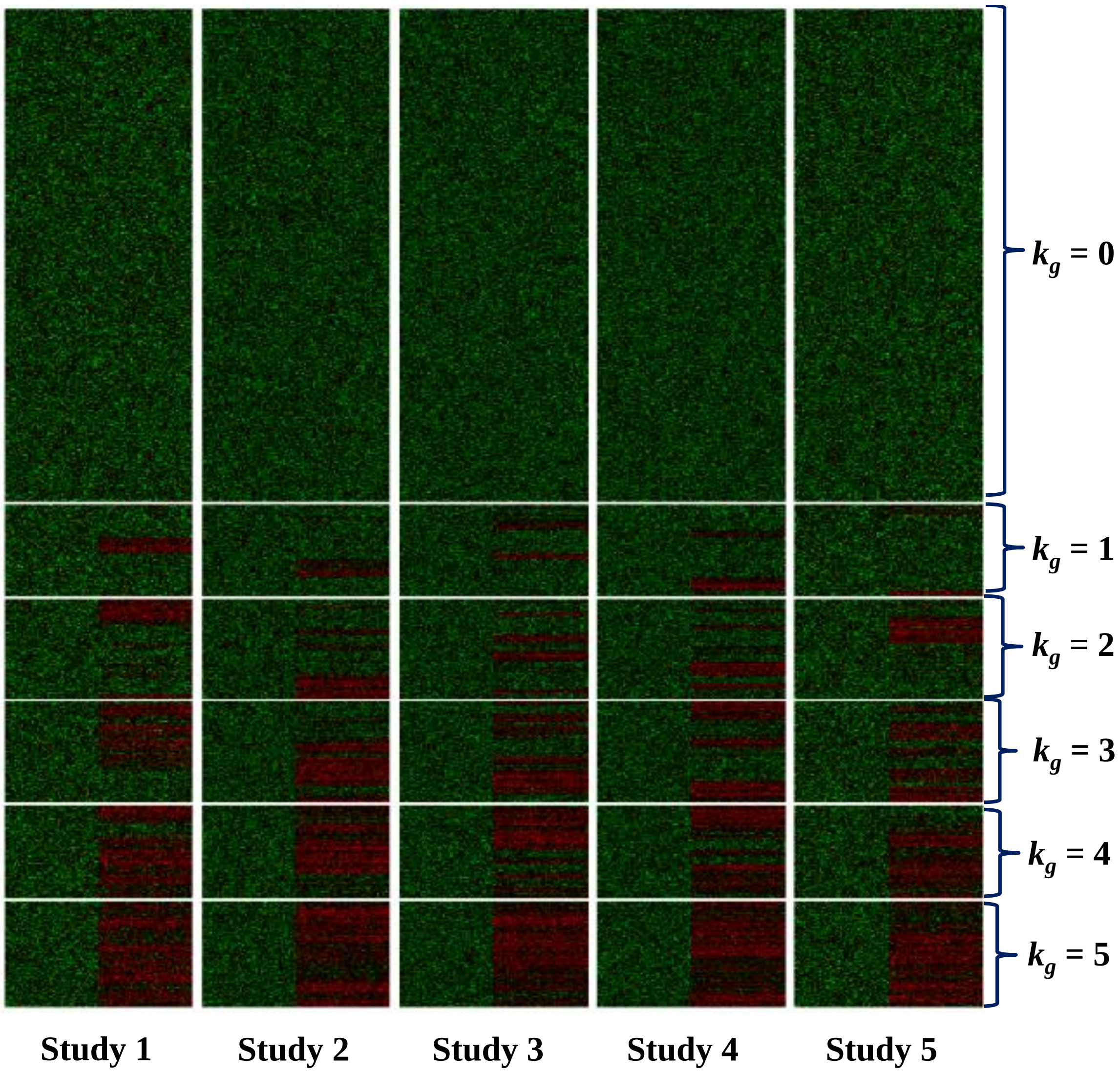

Figure S2: Heatmap of simulated example (red color represents up-regulated genes)

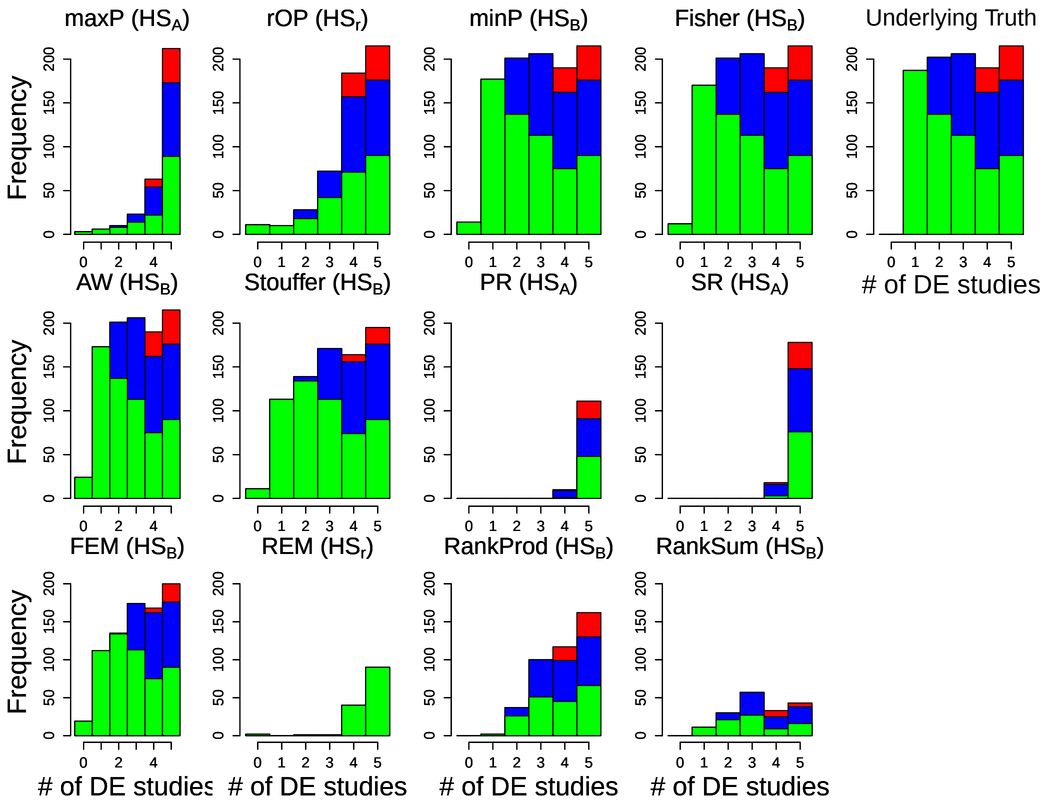

Figure S3: The histograms of the true number of DE studies among detected DE genes under FDR=5% in each method for discordance case (green color represents all concordance effect sizes; blue color represents one study has opposite effect size and red color represents two studies have opposite effect size).

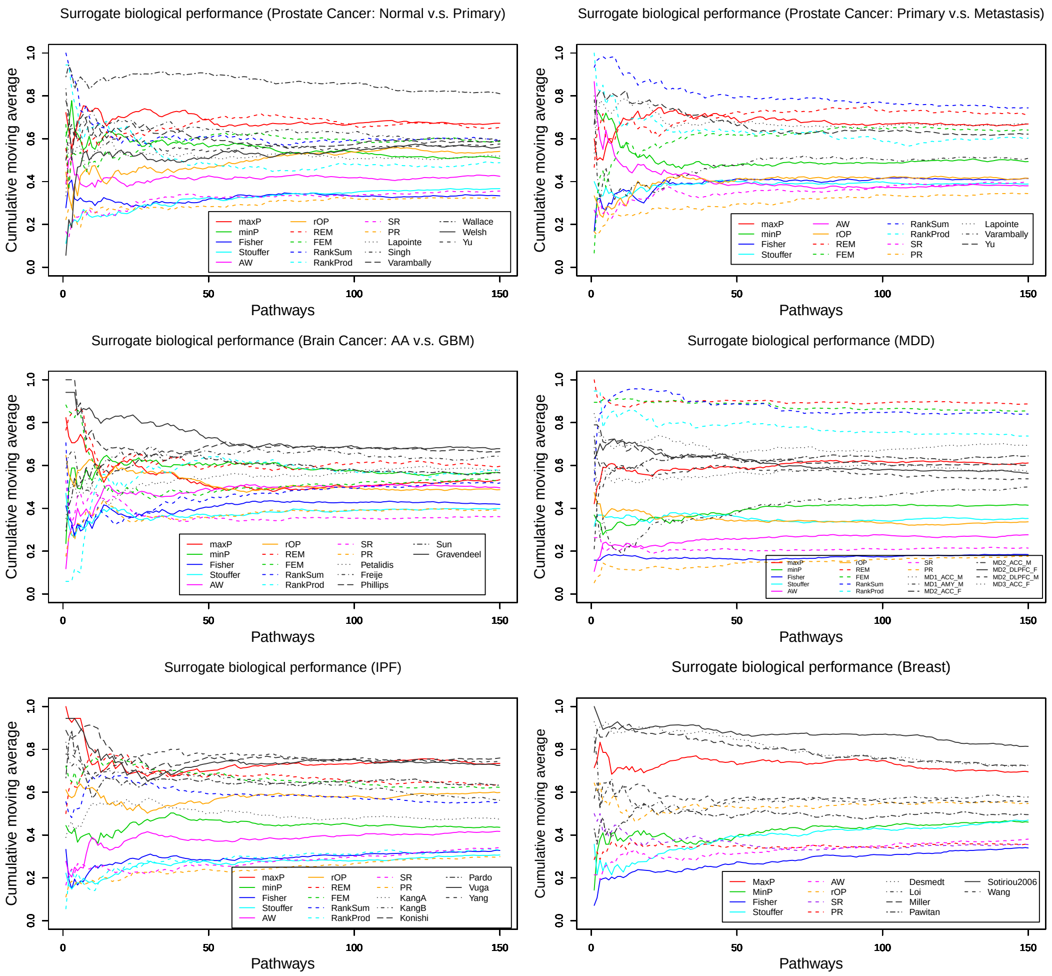

Figure S4: Cumulative moving average to determine $D = 100$

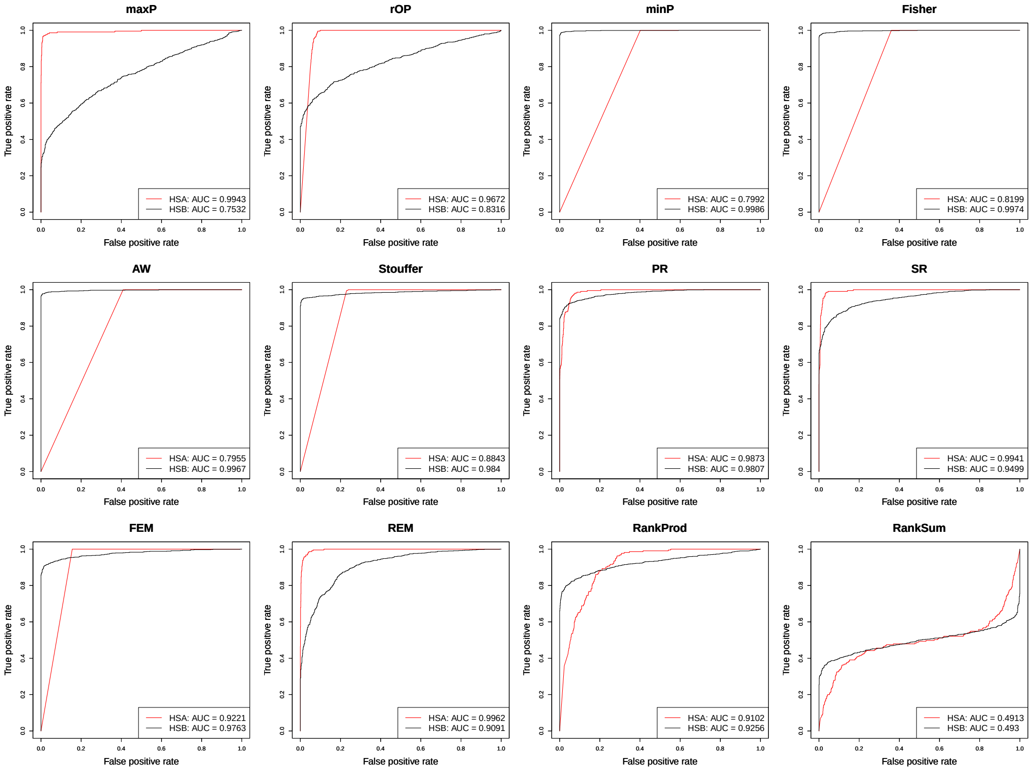

Figure S5: The ROC curves and AUC for the hypothesis settings of HS_A-type and (red line) HS_B-type (black line) in each meta-analysis method

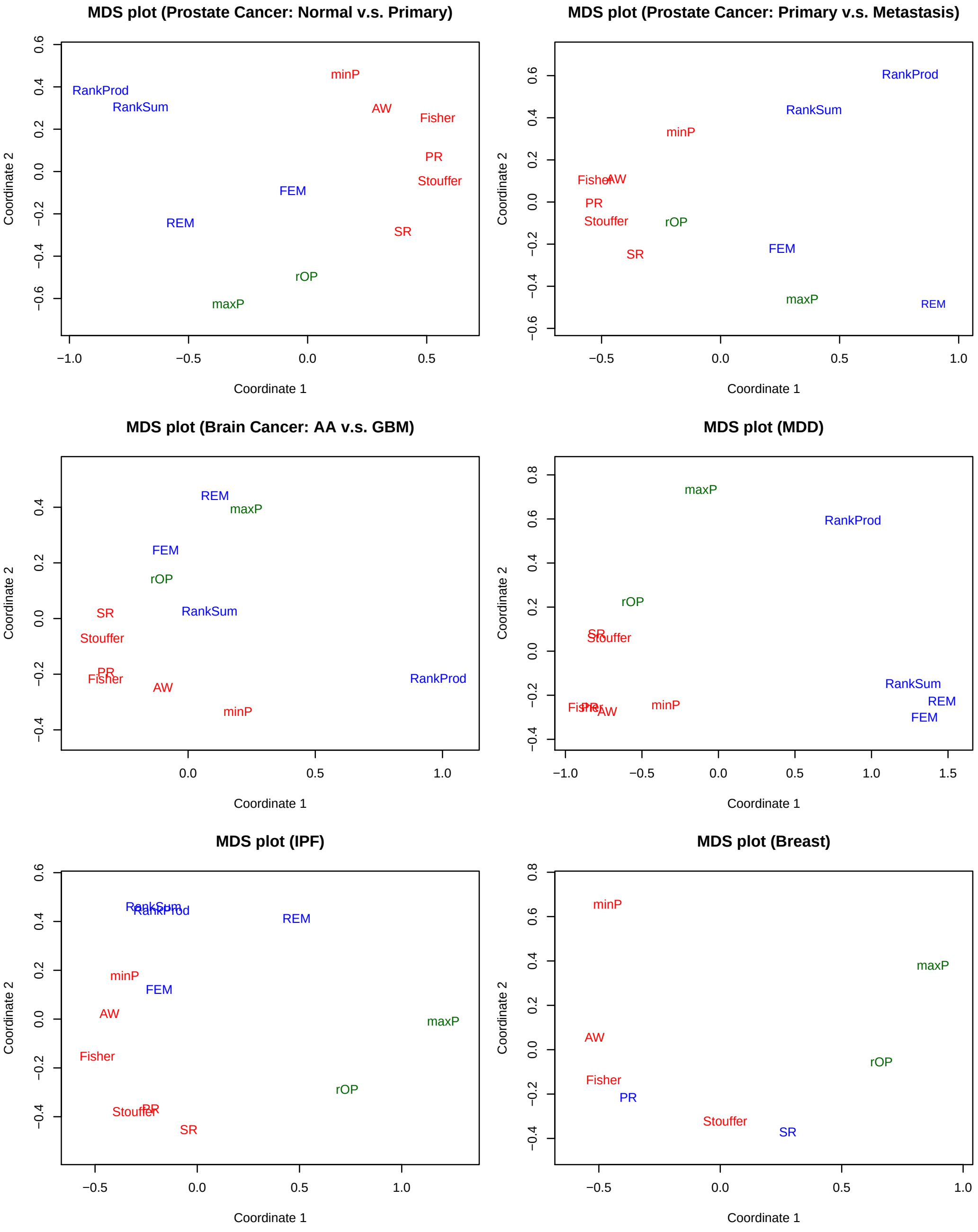

Figure S6: Multidimensional scaling (MDS) plots of individual data sets

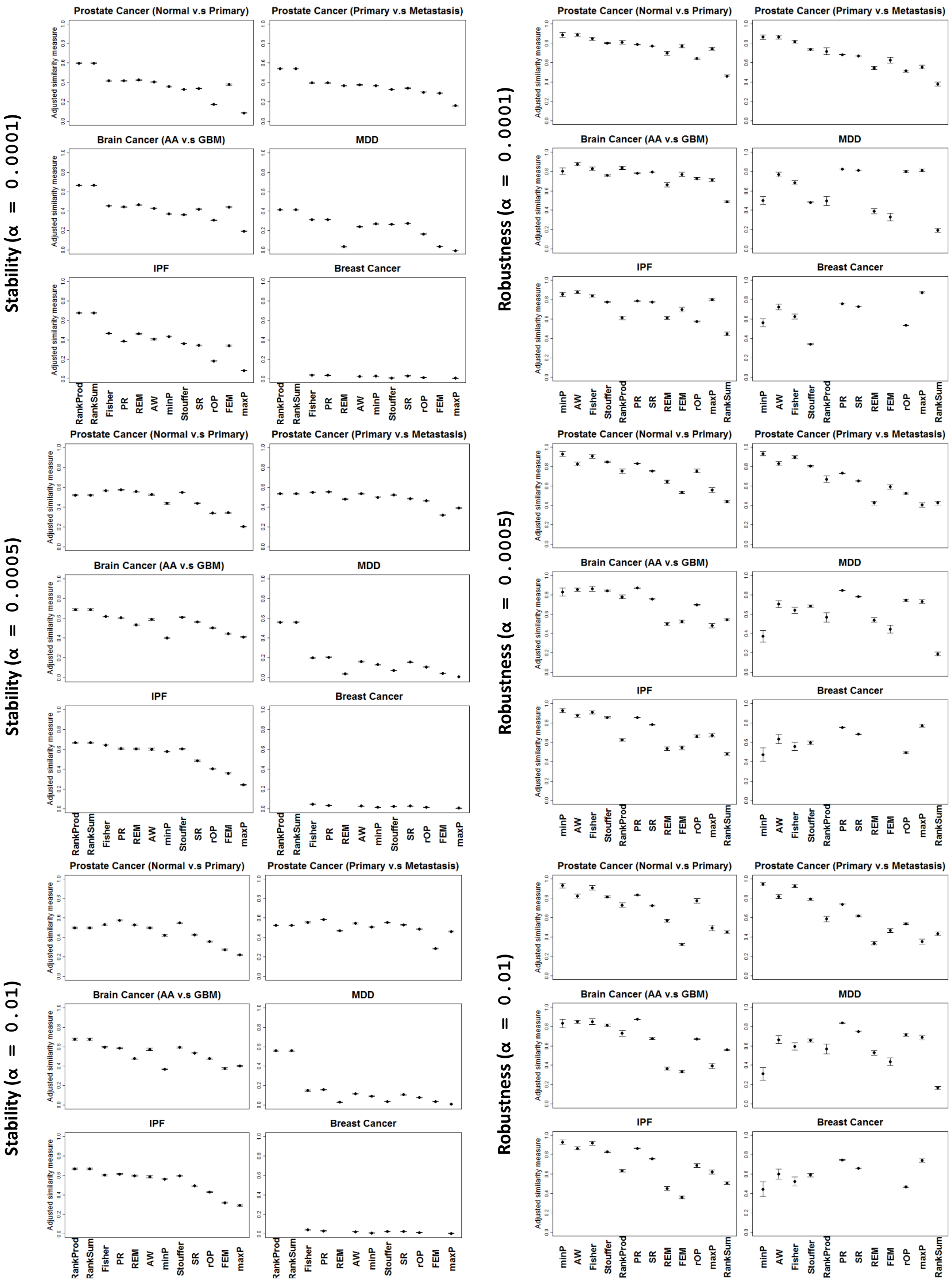

Figure S7: Stability and Robustness plot for $\alpha = 0.0001$, 0.005 and 0.01

Table S1: Detailed data sets description

	Author	Year	Platform	Sample Size (Case/Controls)	Source
Prostate Cancer Studies (Normal v.s Primary)	Welsh	2001	HG-U95A	34(25/9)	public.gnf.org/cancer/
	Singh	2002	HG-U95Av2	102(52/50)	www.broad.mit.edu
	Lapointe	2004	cDNA	103(62/41)	GSE3933
	Yu	2004	HG-U95Av2	83(65/18)	GSE6919
	Varambally	2005	HG-U133 Plus 2	13(7/6)	GSE3325
	Wallace	2008	HG-U133A2	89(69/20)	GSE6956
	Nanni	2006	HG-U133A	30(23/7)	GSE3868
Prostate Cancer Studies (Primary v.s Metastasis	Lapointe	2004	cDNA	71(62/9)	GSE3933
	Varambally	2005	HG-U133 Plus 2	13(7/6)	GSE3325
	Yu	2004	HG-U95Av2	90(65/25)	GSE6919
	Tomlins	2006	cDNA	49(30/19)	GSE6099
Brain Cancer Studies	Freije	2004	HG-U133A,B	85(59/26)	GSE4412
	Phillips	2006	HG-U133A,B	100(76/24)	GSE4271
	Sun	2006	HG-U133 Plus 2	100(81/19)	GSE4290
	Petalidis	2008	HG-U133A	58(39/19)	GSE1993
	Gravendeel	2009	HG-U133 Plus 2	175(159/16)	GSE16011
	Paugh	2010	HG-U133 Plus 2	42(33/9)	GSE19578
	Yamanaka	2006	Agilent	29(22/7)	GSE4381
MDD Studies	MD1_AMY	2009	HG-U133 Plus 2	28(14/14)	Dr. Sibille
	MD1_ACC	2009	HG-U133 Plus 2	32(16/16)	Dr. Sibille
	MD3_ACC	2009	HumanHT-12	44(22/22)	Dr. Sibille
	MD2_ACC_M	2010	HG-U133 Plus 2	18(9/9)	Dr. Sibille
	MD2_ACC_F	2010	HG-U133 Plus 2	26(13/13)	Dr. Sibille
	MD2_DLPFC_M	2010	HG-U133 Plus 2	28(14/14)	Dr. Sibille
	MD2_DLPFC_F	2010	HG-U133 Plus 2	32(16/16)	Dr. Sibille
	MD3_AMY	2009	HumanHT-12	42(21/21)	Dr. Sibille
Lung Disease Studies (IPF)	Pardo	2005	Codelink	24(13/11)	GSE2052
	Yang	2007	Agilent 43K	29(20/9)	GSE5774
	Vuga	2009	Codelink	7(4/3)	GSE10921
	Konishi	2009	Agilent 4x44K	38(23/15)	GSE10667
	KangA	2011	Agilent 4x44K	63(52/11)	Dr. Kaminski
	KangB	2011	Agilent 8x60K	96(75/21)	Dr. Kaminski
	Larsson	2008	HG-U133 Plus 2	12(6/6)	GSE11196
	Emblom	2010	cDNA	58(38/20)	GSE17978
Breast Cancer Studies	Loi	2007	HG-U133A	125	GSE6532
	Miller	2005	HG-U133A,B	236	GSE3494
	Pawitan	2005	HG-U133A,B	159	GSE1456
	Sotiriou2006	2006	HG-U133A	187	GSE2990
	Desmedt	2007	HG-U133A	198	GSE7390
	Wang	2005	HG-U133A	286	GSE2034
	Sotiriou2003	2003	cDNA	110	
	vantVeer	2002	cDNA	97	

Table S2: MetaQC results

Data set	Study	IQC	EQC	CQC_g	CQC_p	AQC_g	AQC_p	Rank
Prostate Cancer Studies (Normal v.s Primary)	1. Welsh	4.38	0.53*	54.63	64.08	18.9	39.09	2.25
	2. Yu	6.64	0.9*	46.91	55.48	14.84	26.2	2.33
	3. Lapointe	2.1*	1.33*	27	53.98	6.28	18.29	3.17
	4. Singh	1.14*	0.95*	14.67	19.21	3.85	18.34	4.17
	5. Varambally	4.38	1.06*	8.7	3.29	2.55	2.41	4.92
	6. Wallace	7.86	0.27*	0*	27.05	0*	3.69	5.33
	7. Nanni	0.75*	0.7*	0.88*	4.2	0.63*	11.45	5.83
Prostate Cancer Studies (Primary v.s Metastasis)	1. Varambally	6.4	0.27*	16.88	23.86	5.5	11.66	1.5
	2. Yu	4.74	0.94*	6.77	13.73	1.43*	6.4	2
	3. Lapointe	3.3	0.8*	2.91	4.08	2.95	5.95	2.67
	4. Tomlins	1.3*	0.51*	0.21*	0.21*	0.1*	0.41*	3.83
Brain Cancer Studies	1. Sun	4.96	2.64	151.63	128.5	61.12	48.82	1.5
	2. Petalidis	4.24	1.17*	148.97	122.39	56.74	75.83	2.83
	3. Freije	5.27	2.52	89.34	68.09	43.31	20.49	3
	4. Phillips	4.81	1.73*	84.93	56	37.22	25.31	3.83
	5. Gravendeel	6.27	1.13*	38.53	48.98	11.9	35.74	4.17
	6. Paugh	1.51*	1.26*	1.62*	0.17*	1.7*	1.77*	6
	7. Yamanaka	0.1*	0.56*	0.92*	0.94*	1.85*	0.31*	6.67
MDD Studies	1. MD2_ACC_F	8.48	1.08*	34.48	54.49	11.9	10.6	1.83
	2. MD2_DLPFC_F	7.87	1.13*	34.58	32.29	6.33	6.91	2.67
	3. MD2_DLPFC_M	2.55	2.08*	24.36	46.97	3.54	20.54	3
	4. MD1_ACC_M	5.03	0.45*	23.25	50.38	4.29	10.74	3.67
	5. MD3_ACC_F	0.74*	1.05*	9.33	9.31	4.8	4.62	5.5
	6. MD2_ACC_M	2.99	1.04*	7.41	9.4	3.36	0.96*	5.83
	7. MD1_AMY_M	1.97*	0.11*	5.47	23.76	1.93*	7.83	6.17
	8. MD3_AMY_F	1.56*	0.96*	0.96*	0.15*	0.38*	2.31	7.33
Lung Disease Studies (IPF)	1. KangA	6.64	0.34*	140.41	85.47	39.01	40.71	2.17
	2. KangB	5.46	0.64*	94.08	45.06	27.4	22.56	2.33
	3. Konishi	6.76	0.77*	17.99	31.45	5.99	21.42	3
	4. Yang	4.07	0.44*	26.61	23.7	9.57	18.41	4.17
	5. Pardo	4.44	0.35*	15.6	29.98	14.56	17.09	4.5
	6. Vuga	2.28	0.39*	1.41*	17.32	1.02*	14.5	6
	7. Larsson	1.85*	1.32*	0.54*	4.83	0.12*	1.26*	6.33
	8. Emblom	0.03*	0.19*	1.68*	0.07*	0.68*	0.56*	7.5
Breast Cancer Studies	1. Pawitan	3.63	4	29.79	116.82	21.99	83.85	2.25
	2. Loi	6.64	4	13.9	66.34	7.32	62.05	2.58
	3. Sotiriou2006	1.3*	0.38*	49.91	134.6	14.3	72.17	3.33
	4. Miller	6.14	4	7.64	47.17	4.24	30.76	3.58
	5. Desmedt	6.26	4	5.09	14.94	3.24	17.21	4.42
	6. Wang	6.03	3.52	0.75*	25.72	2.48	26.01	5.17
	7. Sotiriou2003	2.15*	1.37*	0.28*	4.62	0.07*	2.58	6.83
	8. vantVeer	0.03*	0.26*	0.14*	1.83*	0.15*	0.8*	7.83

Table S3: Data sets and number of matched genes

Disease	# of studies	# of studies passed MetaQC	Comparison	# of matched genes
Prostate cancer	7	6	Binary (normal vs. primary)	6,940
Prostate cancer	4	3	Binary (primary vs. metastastasis)	4,260
Brain cancer	7	5	Binary (AA vs. GBM) (AA vs. GBM)	6,019
Major Deoressive Disorder (MDD)	8	6	Binary (Normal vs. MDD)	6,000
Idiopathic Pulmonary Fibrosis (IPF)	8	6	Binary (Normal vs. IPF)	5,481
Breast cancer	8	6	Survival time (Relapse free survival)	10,688

Based on the QC, the study "Nanni" was removed from 7 prostate cancer studies comparing normal and primary cancer patients; the study "Tomlins" was removed from 4 prostate cancer studies com-paring primary cancer patients and metastasis cancer patients. In the 7 brain cancer studies, the "Paugh" and "Yamanaka" studies were removed. In the case of major depression disorder (MDD) studies, we removed the study "MD3 AMY F". Studies "Larsson" and "Em-blom" were removed from 8 lung disease studies. In breast cancer survival data sets, two cDNA data sets "Sotiriou2003" and "vantVeer" were removed.

Table S4: Mean standardized rank (MSR) and aggregated standardized rank (ASR) for detection capability

	Fisher	AW	Stouffer	minP	FEM	RankSsum	rOP	RankProd	maxP	REM	SR	PR
Prostate cancer (normal v.s. primary)	0.08	0.17	0.25	0.34	0.42	0.51	0.63	0.61	0.78	0.80	0.98	0.93
Prostate cancer (primary v.s. metastasis)	0.08	0.17	0.25	0.33	0.55	0.45	0.51	0.71	0.70	0.83	0.92	1.00
Brain cancer (AA v.s. GBM)	0.08	0.17	0.27	0.40	0.40	0.54	0.57	0.65	0.75	0.75	0.92	1.00
MDD	0.17	0.22	0.26	0.51	0.55	0.48	0.49	0.52	0.72	0.83	0.85	0.90
IPF	0.08	0.17	0.27	0.32	0.42	0.49	0.67	0.60	0.81	0.76	0.97	0.95
Breast Cancer	0.13	0.33	0.41	0.38	NA	NA	0.63	NA	0.75	NA	0.99	0.89
Aggregated standardized ranks	0.11	0.20	0.29	0.38	0.47	0.49	0.58	0.62	0.75	0.79	0.94	0.95

Table S5: Mean standardized rank (MSR) and aggregated standardized rank (ASR) for biological association

	Stouffer	Fisher	AW	PR	rOP	SR	minP	RankProd	FEM	maxP	REM	RankSum
Prostate cancer (normal v.s. primary)	0.42	0.38	0.38	0.41	0.44	0.53	0.49	0.58	0.63	0.69	0.75	0.80
Prostate cancer (primary v.s. metastasis)	0.36	0.38	0.39	0.42	0.36	0.52	0.49	0.55	0.68	0.72	0.78	0.86
Brain cancer (AA v.s. GBM)	0.44	0.45	0.40	0.54	0.42	0.64	0.47	0.50	0.56	0.65	0.72	0.73
MDD	0.27	0.29	0.40	0.28	0.42	0.28	0.56	0.68	0.80	0.84	0.81	0.88
IPF	0.35	0.41	0.36	0.40	0.45	0.48	0.52	0.63	0.66	0.72	0.76	0.78
Breast Cancer	0.45	0.45	0.48	0.42	0.52	0.56	0.71	NA	NA	0.92	NA	NA
Aggregated standardized ranks	0.38	0.39	0.40	0.41	0.43	0.50	0.54	0.59	0.67	0.75	0.76	0.81

Table S6: Mean standardized rank (MSR) and aggregated standardized rank (ASR) for stability

	RankProd	RankSum	Fisher	PR	REM	AW	minP	Stouffer	SR	rOP	FEM	maxP
Prostate cancer (normal v.s. primary)	0.08	0.17	0.40	0.38	0.27	0.46	0.70	0.72	0.71	0.92	0.70	1.00
Prostate cancer (primary v.s. metastasis)	0.08	0.17	0.38	0.43	0.35	0.39	0.58	0.66	0.73	0.84	0.90	1.00
Brain cancer (AA v.s. GBM)	0.08	0.17	0.37	0.40	0.30	0.50	0.74	0.68	0.77	0.67	0.83	1.00
MDD	0.04	0.04	0.23	0.19	0.82	0.45	0.45	0.58	0.36	0.67	0.78	0.90
IPF	0.08	0.17	0.38	0.44	0.32	0.52	0.49	0.61	0.76	0.92	0.82	1.00
Breast Cancer	NA	NA	0.18	0.32	NA	0.50	0.51	0.59	0.70	0.80	NA	0.89
Aggregated standardized ranks	0.07	0.14	0.32	0.36	0.41	0.47	0.58	0.64	0.67	0.80	0.81	0.97

Table S7: Mean standardized rank (MSR) and aggregated standardized rank (ASR) for robustness

	minP	AW	Fisher	Stouffer	RankProd	PR	SR	REM	FEM	rOP	maxP	RankSum
Prostate cancer (normal v.s. primary)	0.20	0.27	0.23	0.34	0.56	0.52	0.54	0.59	0.65	0.80	0.79	1.00
Prostate cancer (primary v.s. metastasis)	0.17	0.21	0.24	0.33	0.47	0.53	0.65	0.65	0.70	0.81	0.79	0.94
Brain cancer (AA v.s. GBM)	0.24	0.29	0.31	0.40	0.52	0.54	0.59	0.63	0.57	0.76	0.71	0.94
MDD	0.55	0.51	0.56	0.59	0.33	0.47	0.37	0.55	0.52	0.49	0.58	0.97
IPF	0.21	0.31	0.29	0.35	0.47	0.49	0.62	0.51	0.74	0.76	0.73	1.00
Breast Cancer	0.57	0.60	0.62	0.51	NA	0.49	0.62	NA	NA	0.40	0.70	NA
Aggregated standardized ranks	0.32	0.37	0.37	0.42	0.47	0.51	0.57	0.59	0.64	0.67	0.72	0.97
